# Aberrantly low STAT3 and STAT5 responses are associated with poor outcome and an inflammatory gene expression signature in pediatric acute myeloid leukemia

**DOI:** 10.1007/s12094-021-02621-w

**Published:** 2021-05-04

**Authors:** P. Narayanan, T.-K. Man, R. B. Gerbing, R. Ries, A. M. Stevens, Y.-C. Wang, X. Long, A. S. Gamis, T. Cooper, S. Meshinchi, T. A. Alonzo, M. S. Redell

**Affiliations:** 1 Texas Children’s Cancer Center, Baylor College of Medicine, Houston, TX USA; 2 Children’s Oncology Group, Monrovia, CA USA; 3 Fred Hutchinson Cancer Research Center, Seattle, WA USA; 4 Children’s Mercy Hospital and Clinics, Kansas, MO USA; 5 Seattle Children’s Hospital, Seattle, WA USA; 6 Division of Biostatistics, University of Southern California, Los Angeles, CA USA

**Keywords:** Pediatric AML, STAT3, STAT5, Microenvironment, Bone marrow stroma, Inflammation

## Abstract

The relapse rate for children with acute myeloid leukemia is nearly 40% despite aggressive chemotherapy and often stem cell transplant. We sought to understand how environment-induced signaling responses are associated with clinical response to treatment. We previously reported that patients whose AML cells showed low G-CSF-induced STAT3 activation had inferior event-free survival compared to patients with stronger STAT3 responses. Here, we expanded the paradigm to evaluate multiple signaling parameters induced by a more physiological stimulus. We measured STAT3, STAT5 and ERK1/2 responses to G-CSF and to stromal cell-conditioned medium for 113 patients enrolled on COG trials AAML03P1 and AAML0531. Low inducible STAT3 activity was independently associated with inferior event-free survival in multivariate analyses. For inducible STAT5 activity, those with the lowest and highest responses had inferior event-free survival, compared to patients with intermediate STAT5 responses. Using existing RNA-sequencing data, we compared gene expression profiles for patients with low inducible STAT3/5 activation with those for patients with higher inducible STAT3/5 signaling. Genes encoding hematopoietic factors and mitochondrial respiratory chain subunits were overexpressed in the low STAT3/5 response groups, implicating inflammatory and metabolic pathways as potential mechanisms of chemotherapy resistance. We validated the prognostic relevance of individual genes from the low STAT3/5 response signature in a large independent cohort of pediatric AML patients. These findings provide novel insights into interactions between AML cells and the microenvironment that are associated with treatment failure and could be targeted for therapeutic interventions.

**Supplementary Information:**

The online version contains supplementary material available at 10.1007/s12094-021-02621-w.

## Background

Children with acute myeloid leukemia (AML) are treated with aggressive regimens that often include stem cell transplantation, yet event-free survival (EFS) rates remain approximately 60% [1, 2]. Current risk assignment algorithms incorporate cytogenetics, various somatic mutations, and residual disease after one cycle of chemotherapy [3]. This strategy misses a substantial fraction of patients who need more intensive therapy, because 20–30% of “low-risk” patients relapse [1, 4]. At the other end of the spectrum, 25–30% of high-risk patients who are transplanted in first remission die of relapse or toxicity [5, 6]. Our goal is to define AML-microenvironment interactions that contribute to relapse and can be targeted pharmacologically.

Signal transducer and activator of transcription 3 (STAT3) and STAT5 mediate signals from hematopoietic factors in the bone marrow, including G-CSF, GM-CSF, IL-6, IL-3, and others. In general, these signals promote proliferation and survival. Aberrant STAT3/5 activity is found in numerous malignancies and confers resistance to chemotherapy and targeted inhibitors [7–9]. In studies of AML specimens from adults, robust activation of STAT3 and STAT5 is associated with induction failure [10, 11]. In children, however, weak activation of STAT3 is associated with inferior EFS [12, 13]. This difference between adult and pediatric AML at the functional level is consistent with many differences at the molecular level [14]. Therefore, efforts to develop prognostic biomarkers and targeted therapies need to be informed by the unique biology of the pediatric disease.

Here, we evaluated signaling responses and gene expression profiles for patients who were treated on the Children’s Oncology Group trials AAML03P1 and AAML0531. We quantified signaling responses to individual factors and to HS5 stromal cell-conditioned medium (CM). Since HS5 cells secrete dozens of hematopoietic factors, CM more closely recapitulates the bone marrow soluble factor milieu. We found that STAT3 and STAT5 responses were associated with EFS in this contemporary patient population. Further, we identified genes that were differentially expressed between cases that did and did not activate these pathways. These genes suggest biological features that may contribute to relapse and represent therapeutic opportunities.

## Methods

### Cells

Cryopreserved bone marrow specimens from 159 pediatric patients with de novo AML were obtained from the Children’s Oncology Group (COG) Biopathology Center, and 6 from the Texas Children’s Hospital Research Tissue Support Service. All COG patients were enrolled on the treatment protocols AAML03P1 or AAML0531. Patient families gave written informed consent, in accordance with the Declaration of Helsinki, for banking of bone marrow for future research. The treatment regimens and outcomes for these trials have been reported [1, 15]. Bone marrow samples were enriched for mononuclear cells by density centrifugation and cryopreserved. These studies were approved by the Institutional Review Board of Baylor College of Medicine.

The human AML cell line Kasumi-1 served as the positive control for flow cytometry studies. Kasumi-1 cells were obtained from ATCC and grown in RPMI 1640 (ATCC) with 10% fetal bovine serum (FBS; Invitrogen), 2 mM L-glutamine, 100 units/ml penicillin and 100 μg/ml streptomycin (pen/strep; Invitrogen). The HS5 cell line was purchased from ATCC and maintained in DMEM with 10% FBS, 2 mM L-glutamine and pen/strep. Kasumi-1 and HS5 cells were grown in a humidified 37 °C incubator with 5% CO_2_. For HS5-conditioned medium (CM), HS5 cells were seeded at 10^5^ cells/ml and allowed to attach overnight. Medium was then replaced with RPMI + additives and collected after 48 h. CM was centrifuged to remove cells and stored at − 20 °C. CM stocks were thawed only once. Cell lines were authenticated by STR profiling annually.

### Flow cytometry

**Fig. 1:**
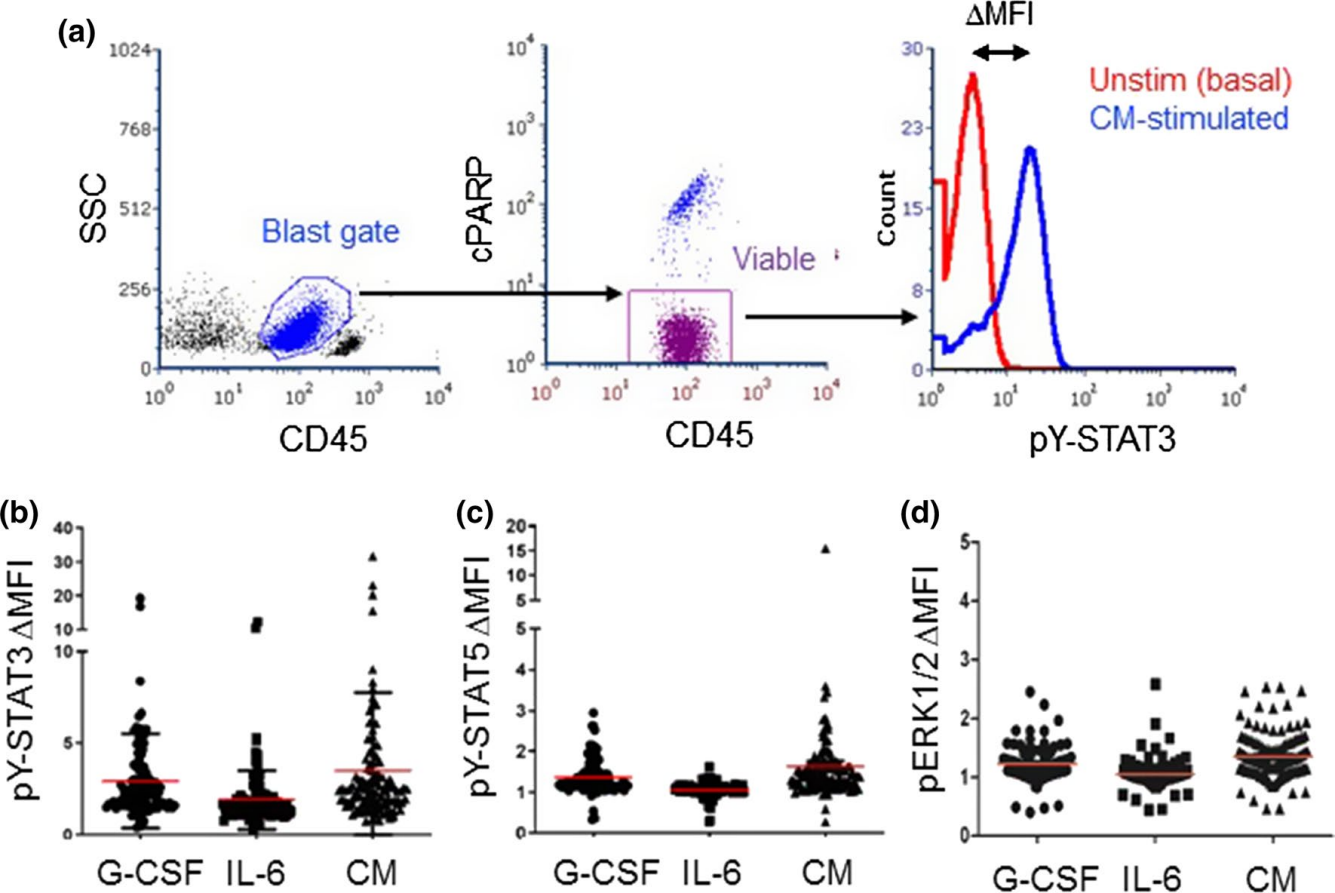
Pediatric AML samples showed variable pY-STAT3 and pY-STAT5 responses to stimulation. **a** The gating strategy involved isolation of blasts and exclusion of lymphocytes based on CD45 v. SSC, followed by exclusion of non-viable (cPARP +) cells. Signaling pathway activation was quantified for viable blasts. The ΔMFI was calculated as the MFI for stimulated cells/MFI for unstimulated cells. **b**–**d** Primary pediatric AML samples were stimulated with G-CSF (10 ng/ml; *n* = 111), IL-6 + sIL-6R (5 ng + 10 ng/ml; *n* = 111), 50% CM (*n* = 113) or vehicle. Activated pY-STAT3, pY-STAT5, and pERK1/2 were measured by flow cytometry. The red bar indicates the mean

Samples were thawed and evaluated for viability as previously described [13]. Only samples with at least 60% viability and sufficient cell number were processed. Ultimately, 122 COG samples and 4 local samples were suitable for analysis. Cells were distributed at 2–3 × 10^5^ cells/well for stimulation; control wells remained unstimulated. Stimulation with individual factors included G-CSF (10 ng/ml); IL-6 + soluble IL-6 receptor α (sIL-6R; 5 + 10 ng/ml, respectively); GM-CSF (1 ng/ml); and IL-3 (5 ng/ml). G-CSF (filgrastim) was purchased from the Texas Children’s Hospital Pharmacy and the others were from R&D Systems. For CM stimulation, 0.5 ml CM was added to AML cells suspended in 0.5 ml fresh medium, to yield 50% CM. After 15 min, cells were fixed, permeabilized, and stained for FACS as described [16]. Antibodies for flow cytometry were purchased from BD Biosciences unless otherwise noted, and included CD45-APC-H7 (clone 2D1); cleaved PARP (cPARP)-AlexaFluor 647 or AlexaFluor 700 (clone F21-852); pY-STAT3-PE (clone 4/P); pY-STAT5-PerCP-Cy5.5 or -AlexaFluor 647 (clone 47); pY-ERK1/2-Pacific Blue (clone 197G2; Cell Signaling Technologies); CD116-BV421 (clone M1); CD123-APC (clone 7G3); and CD131-AlexaFluor 647 (clone 3D7). Data were acquired with Diva on a customized 5-laser LSRII (BD) and analyzed with FCS Express v6 (DeNovo). Gating steps isolated viable blasts, based on FSC v SSC, CD45^dim^, and cPARP^neg^ (Fig. 1a).

### Gene expression analyses

Existing RNA-sequencing (RNA-seq) databases were mined for this study. The RNA-seq count data were imported, normalized and analyzed using the RNA-seq function of BRB ArrayTools [17]. Unreliable low-count transcripts in all samples were filtered. Then, a differential expression analysis of the specified STAT3/5 response classes was performed based on the edgeR quasi-likelihood pipeline with sample batch correction [18]. The genes were called significant at false discovery rate (FDR) = 0.05. Upstream regulator and pathway analyses were conducted using Ingenuity Pathway Analysis software (Qiagen). The upstream regulator analysis is based on prior knowledge of expected effects between regulators and targets in the Ingenuity Knowledge Base. The analysis identified the targets of different regulators in the RNA-seq dataset and compared the direction of change of a target gene with the expected change in the literature. Then, activation *z*-score is calculated to infer predicted activation or inactivation states of the upstream regulators relative to the random assignments. Activation *z*-score >  = 2 is called activated. The overlap *p*-value evaluates if the overlap between the differentially expressed genes and known targets regulated by a regulator is significant.

### Statistics

Statistical analysis of signaling data was performed using Prism 5.02 (GraphPad Software Inc., La Jolla, CA, USA). Values are means ± standard error of the mean (SEM). A two-tailed Pearson correlation analysis was used to assess the degree of relationship between two continuous variables.

Clinical data were analyzed through September 30, 2015 for AAML03P1 patients and through June 30, 2019 for AAML0531 patients. The significance of observed differences in proportions was tested using the Chi-squared test and Fisher’s exact test when data were sparse. Comparisons of medians were tested using the Kruskal–Wallis test. Five-year estimates of event-free (EFS) and overall survival (OS) from study entry were calculated by the Kaplan–Meier method. Estimates are reported with two times the Greenwood standard error. OS is defined as time from study entry to death from any cause. EFS is defined as time from study entry to induction failure, relapse, or death from any cause. EFS and OS were compared for significant differences by the log-rank test. The Cox proportional hazards model was used to estimate hazard ratios (HR) and to determine the prognostic value of the ligand response categories. Exploratory multiple cutpoint analyses were performed by estimating the HR for OS and EFS by examining all cutpoints between 10 and 90% quantile levels of each marker and comparing the lower versus higher groups for each cutpoint.

Analyses of the relationships between gene expression levels and EFS were conducted for 1061 patients enrolled on AAML1031, with clinical data through March 31, 2020. RNA-seq data in transcripts per million (TPM) were available from the TpAML project. Cases were separated into quartiles and outcomes compared as described above.

## Results

### A subset of pediatric AML cases failed to activate STAT3/5 signaling in response to multiple stimuli

We studied diagnostic specimens from pediatric AML patients enrolled on the COG trials AAML03P1 or AAML0531. Both studies used the same chemotherapy backbone. Patients on AAML03P1 and AAML0531 arm B also received gemtuzumab ozogamicin [1, 15]. The 5-year EFS and OS for the 113 patients with both HS5 CM response data and outcome data were 50.9 ± 9.6% and 65.6 ± 9.2%, respectively. The 5-year EFS and OS for all other eligible patients on these trials (*n* = 1248) were 49.2 ± 2.9% and 63.9 ± 2.8%, respectively. These survival rates are not significantly different, confirming that the study cohort is clinically representative of the pediatric AML population.

Most prior studies of signaling responses in leukemia cells used single factors. To more accurately represent the microenvironment, we stimulated AML cells with HS5 CM. Previously, we quantified soluble factors in both HS5 CM [19] and bone marrow plasma from pediatric AML patients [20]. The factor levels present in 50% CM are within the range of levels in AML bone marrow plasma for 32 of 41 factors (Online Resource Table S1), including IL-3, IL-6, G-CSF, and GM-CSF.

We quantified the signaling responses to G-CSF, IL-6 and 50% CM using our established flow cytometry assay. We evaluated phosphotyrosine-STAT3 (pY-STAT3) and pY-STAT5, given their anti-apoptosis effects and associations with patient outcome [10]. For comparison, we also evaluated pERK1/2 (pT202/pY204) responses to the same stimuli. We quantified responses as the fold change in mean fluorescence intensity for the stimulated condition over the unstimulated condition (∆MFI, Fig. 1a). Figure 1b–d illustrates the ranges of inducible pY-STAT3, pY-STAT5, and pERK1/2 responses.

**Fig. 2:**
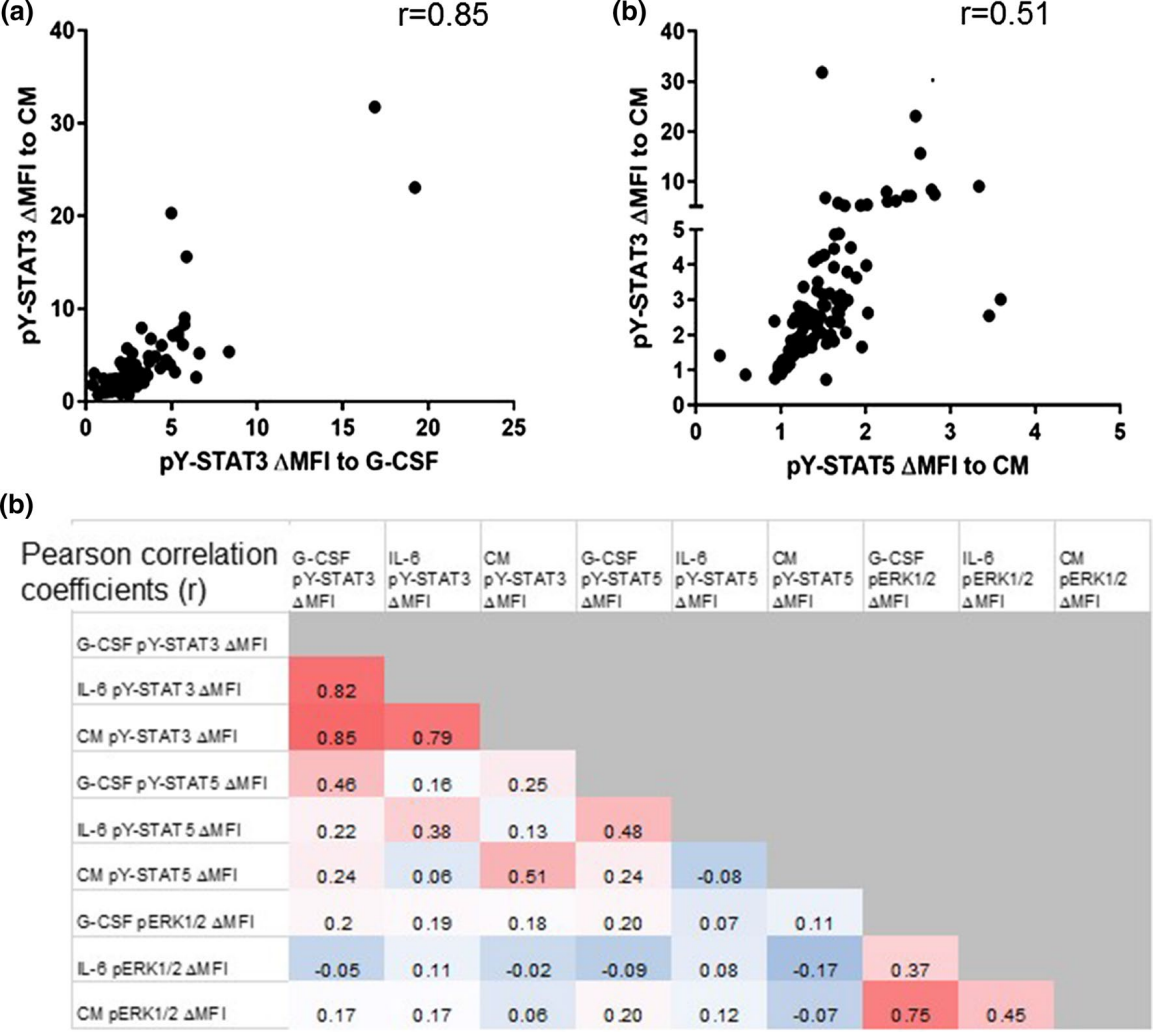
Ligand-induced pY-STAT3 and pY-STAT5 responses were strongly correlated. **a** The pY-STAT3 ΔMFIs in response to G-CSF and CM were strongly correlated. (*n* = 111), as were the pY-STAT3 and pY-STAT5 ΔMFIs in response to CM stimulation (*n* = 113). *r* = Pearson correlation coefficient. **b** The heatmap illustrates the degree of correlation for each ligand-induced signaling parameter with the others. Numbers represent the Pearson correlation coefficient (*r*)

Pearson correlations of ∆MFIs revealed that each signaling parameter responded similarly to different stimuli (Fig. 2). The pY-STAT3 responses were especially consistent between different stimuli, with correlation coefficients 0.79–0.85 (*p* < 0.0001). A sample that strongly activated STAT3 with G-CSF also strongly activated STAT3 with IL-6 and CM, whereas samples that failed to increase pY-STAT3 in response to individual ligands also failed to respond to CM. Interestingly, the correlation between G-CSF-activated pY-STAT5 and CM-activated pY-STAT5 was weak (*r* = 0.24), suggesting that CM contains factors that activate STAT5 independently of its activation by G-CSF. The pY-STAT3 and pY-STAT5 responses to a given stimulus were moderately correlated (Pearson *r* = 0.38–0.51), and a subset of samples responded poorly in both parameters.

Given the variability between G-CSF-induced pY-STAT5 and CM-induced pY-STAT5, we measured responses to GM-CSF and IL-3 in 13 additional samples. GM-CSF and IL-3 activate pY-STAT5 but not pY-STAT3, and they use the same CD131 co-receptor. The GM-CSF- and IL-3-induced pY-STAT5 responses were strongly correlated with a Pearson coefficient of 0.75 (Online Resource Fig. S1). The pY-STAT5 responses to GM-CSF and IL-3 were weakly correlated with pY-STAT5 responses to CM. We again noted cases that failed to activate pY-STAT5 downstream of multiple stimuli. Therefore, while physiological STAT5 activity reflects multiple upstream ligands, a subset of cases remains uniformly unresponsive.

To determine whether samples that failed to active STAT3/5 pathways also failed to activate a mechanistically different pathway, we also measured inducible pERK1/2 (Fig. 1d) [12, 21]. Each sample’s pERK1/2 responses to the different stimuli were positively correlated (Pearson *r* = 0.37–0.75), and there were samples that failed to activate pERK1/2 in response to any stimuli. However, pERK1/2 ∆MFIs did not correlate with pY-STAT3 or pY-STAT5 downstream of any of the stimuli (Fig. 2b), indicating that samples with failed STAT3 or STAT5 activation could still activate ERK1/2, and vice versa. Therefore, the mechanism(s) accounting for dysfunctional STAT3/5 signaling are likely different from those underlying dysfunctional pERK1/2 signaling.

### Failure to respond to stimulation was not related to basal activation or receptor expression

One possible explanation for aberrant pY-STAT3/5 activation is that the pathway is highly constitutively activated, precluding further inducible activation. Unstimulated pY-STAT3 and pY-STAT5 levels were generally low and only weakly inversely correlated with the ligand-induced ∆MFI (Online Resource Fig. S2a), arguing against this explanation. Another possible explanation for failed signaling is low receptor expression. Previously, we showed that G-CSF-induced pY-STAT3 was positively correlated with G-CSF receptor expression, but IL-6-induced pY-STAT3 was inversely correlated with gp130 expression [13]. Here, we measured the expression of CD116 and CD123, the alpha receptor subunits for GM-CSF and IL-3, respectively, and their common beta receptor subunit, CD131, for the same 13 samples with which these ligand responses were evaluated (Online Resource Fig. S2b). Expression of CD116 and CD123 were variable and not related to ligand-induced pY-STAT5 (Online Resource Fig. S2c-d). CD131 surface expression was uniformly low, as has been reported [22]. Therefore, the signaling dysfunction noted in a subset of pediatric AML cells cannot be attributed to elevated constitutive activation nor to absence of surface receptors.

### Environment-induced pY-STAT3 and pY-STAT5 were associated with EFS

**Fig. 3:**
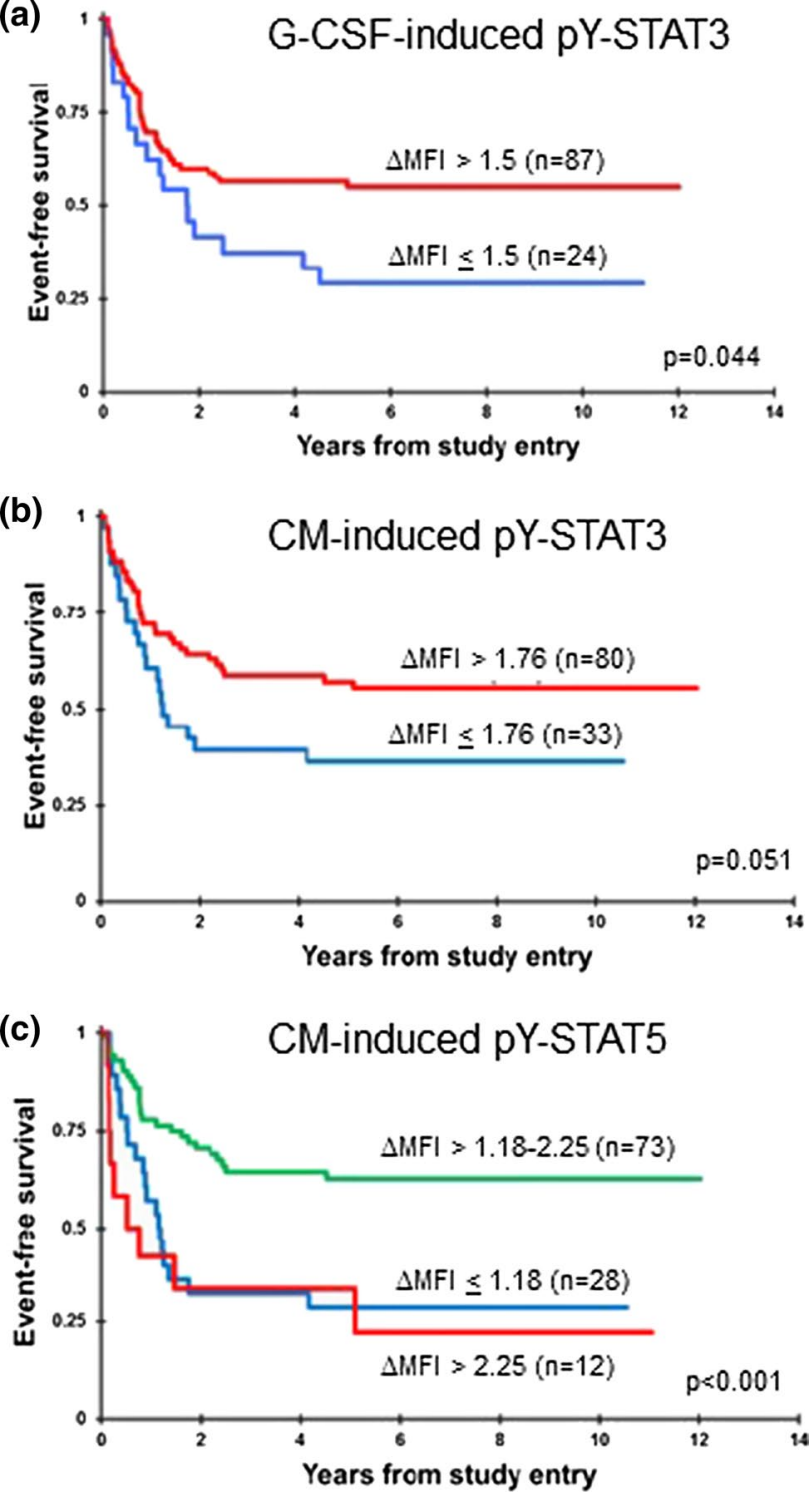
Inducible pY-STAT3 and pY-STAT5 were associated with outcome. **a** Kaplan–Meier survival curves show that patients whose AML cells had a G-CSF-induced pY-STAT3 ΔMFI ≤ 1.5 (*n* = 24) had a 5-year EFS of 29.2 ± 18.6%, compared to 56.4 ± 10.8% for patients with a ΔMFI > 1.5 (*n* = 87; *p* = 0.044). **b** Patients whose AML cells had a CM-induced pY-STAT3 ΔMFI ≤ 1.76 (*n* = 33) had a 5-year EFS of 36.4 ± 16.7%, compared to those whose AML cells responded above this threshold (*n* = 80; 57.2 ± 11.4%, *p* = 0.051). **c** Patients with CM-induced pY-STAT5 MFI ≤ 1.18 (*n* = 28) and patients with CM-induced pY-STAT5 MFI > 2.25 (*n* = 12) had 5-year EFS of 28.6 ± 17.1% and 33.3 ± 27.2%, respectively, compared to the patients in the intermediate-response group (*n* = 73; 62.8 ± 11.6%; *p* < 0.001)

In our analysis of samples from patients treated on CCG2961, patients whose AML cells had a G-CSF-induced pY-STAT3 ∆MFI < 2 had an inferior EFS compared to patients with pY-STAT3 ∆MFI ≥ 2 [13]. We first independently validated this association in pediatric AML patients treated with contemporary regimens. We performed cutpoint analyses to identify a threshold that best discriminated patients with good and poor outcomes. For G-CSF-induced pY-STAT3 (Fig. 3a), patients whose AML cells had a ∆MFI ≤ 1.5 had an inferior EFS (*n* = 24; 5-year EFS 29.2 ± 18.6%) compared to patients whose AML cells had a ∆MFI > 1.5 (*n* = 87; 5-year EFS 56.4 ± 10.8%; *p* = 0.044). This result confirms our prior observation with a similar cutpoint. There was no significant difference in OS between groups (54.2 ± 20.3% v. 68.4 ± 10.3%; *p* = 0.244; Online Resource Fig. S3a). IL-6-induced pY-STAT3 was not associated with EFS in this cohort, nor in the CCG2961 group.

For CM-induced pY-STAT3, patients whose AML cells had ∆MFI ≤ 1.76 (*n* = 33) had a 5-year EFS of 36.4 ± 16.7% (Fig. 3b), compared to the 80 patients whose AML cells responded above this threshold (57.2 ± 11.4%, *p* = 0.051). The OS did not differ significantly between groups (57.1 ± 17.4% v. 69.2 ± 10.8%; *p* = 0.172; Online Resource Fig. S3b). The relationship between inducible pY-STAT5 and EFS was more complex. We did not find an association between G-CSF-induced pY-STAT5 and EFS in this study or previously. For CM-induced pY-STAT5, the cutpoint analysis discriminated three groups (Fig. 3c). Patients with failed pY-STAT5 (∆MFI ≤ 1.18, *n* = 28) and patients with robust pY-STAT5 (∆MFI > 2.25, *n* = 12) had poorer survival rates (5-year EFS of 28.6 ± 17.1% and 33.3 ± 27.2%, respectively), compared to patients in the intermediate-response group (*n* = 73; 5-year EFS 62.8 ± 11.6%; *p* < 0.001). The 5-year OS was 45.5 ± 19.1% for the low-response group, 58.3 ± 28.5% for the high-response group, and 75.0 ± 10.6% for the intermediate-response group (*p* = 0.009; Online Resource Fig. S3c). These results demonstrate that aberrant ligand-induced STAT3 or STAT5 signaling identifies patients with poor EFS.

### STAT3/5 signaling patterns were associated with age and core binding factor fusions

The low pY-STAT3/5 response groups were enriched for infants age < 2 yr (Online Resource Tables S2–S3). This finding is interesting because infants have different cytogenetic fusions than patients in older age groups [14]. As has been reported [13, 23], the higher pY-STAT3 response groups were enriched for patients with core binding factor fusions *t*(8;21) and inv(16) (Online Resource Table S2), which confer a favorable prognosis. We also saw enrichment of M2 morphology in the high pY-STAT3 response groups, suggesting that the level of differentiation may be related to the signaling phenotype [24, 25]. Almost half of the patients in the highest STAT5 response category had FLT3/ITD (Online Resource Table S3). The association between FLT3/ITD, STAT5 activity, and poor outcome is well established [26, 27], although the contribution of environment-induced pY-STAT5 to poor survival in this group of patients has not been elucidated.

Univariable Cox models for EFS supported the results of the Kaplan–Meier comparisons (Table 1). Patients with low G-CSF-induced pY-STAT3 and low CM-induced pY-STAT3 had approximately a 70% higher risk of having an event (induction failure, relapse, or death) compared to those with higher G-CSF and CM-induced pY-STAT3 (*p* = 0.047 and 0.054, respectively). Patients with low CM-induced pY-STAT5 were more than twice as likely to have an event (*p* = 0.001), and those with the highest CM-induced pY-STAT5 were more than three times as likely to have an event (*p* = 0.001), compared to patients with CM-induced pY-STAT5 responses in the intermediate range. Only low CM-induced pY-STAT5 response was a significant factor for OS. For relevant clinical variables, patients with low-risk cytomolecular features [*t*(8;21), inv(16), CEBPα mutation, NPM1 mutation] had HRs below 0.3 for EFS and OS (*p* < 0.001), while age and presenting WBC were not significant factors.

**Table 1:** Univariable cox models

Univariable							
	*N*	EFS from study entry			OS from study entry		
		HR	95% CI	*p*	HR	95% CI	*p*
G-CSF-induced pY-STAT3 ∆MFI							
> 1.5	87	1			1		
< = 1.5	24	1.79	1.01–3.17	0.047	1.52	0.75–3.07	0.247
CM-induced pY-STAT3 ∆MFI							
> 1.76	80	1			1		
< = 1.76	33	1.71	0.99–2.95	0.054	1.58	0.81–3.08	0.176
CM-induced pY-STAT5 ∆MFI							
1.18–2.25	73	1			1		
< = 1.18	28	2.61	1.45–4.70	0.001	2.79	1.39–5.59	0.004
> 2.25	12	3.45	1.61–7.38	0.001	2.25	0.83–6.09	0.113
Age at dx (in years)							
> = 2	103	1			1		
0–1	10	0.93	0.37–2.34	0.881	0.85	0.26–2.76	0.785
WBC (× 10^3^/MicroL)							
≤ 100	89	1			1		
> 100	24	1.59	0.88–2.87	0.128	0.80	0.35–1.81	0.585
Cytomolecular risk							
Standard	30	1			1		
Low	63	0.29	0.16–0.53	< 0.001	0.23	0.11–0.51	< 0.001
High	18	1.23	0.63–2.42	0.546	0.79	0.36–1.77	0.571

The multivariable models for EFS and OS combining each STAT response group with age, presenting WBC and cytomolecular risk group are presented in Table 2. Low CM-induced pY-STAT3 was a significant adverse factor (*p* = 0.028). CM-induced pY-STAT5 retained strong prognostic value with event HRs > 3 for the low- and high-response groups (*p* = 0.001). A low CM-induced pY-STAT5 response was also an adverse factor for OS (*p* = 0.008). Therefore, impaired environment-induced STAT3/5 activation is a clinically relevant phenotype that can identify pediatric AML patients at higher risk of treatment failure, independent of cytomolecular features.

**Table 2:** Multivariable cox models

Multivariable with G-CSF-induced pY-STAT3							
	*N*	EFS from study entry			OS from study entry		
		HR	95% CI	*p*	HR	95% CI	*p*
G-CSF-induced pY-STAT3 ∆MFI							
> 1.5	85	1			1		
< = 1.5	24	1.43	0.77–2.66	0.257	1.13	0.54–2.39	0.744
Age at dx (in years)							
> = 2	99	1			1		
0–1	10	0.34	0.12–0.98	0.046	0.44	0.12–1.61	0.214
WBC (× 10^3^/MicroL)							
≤ 100	86	1			1		
> 100	23	1.87	0.93–3.75	0.078	0.74	0.28–1.95	0.538
Cytomolecular risk							
Standard	29	1			1		
Low	62	0.21	0.10–0.42	< 0.001	0.20	0.09–0.46	< 0.001
High	18	0.75	0.33–1.67	0.478	0.69	0.27–1.73	0.427
Multivariable with CM-induced pY-STAT3							
CM-induced pY-STAT3 ∆MFI							
> 1.76	78	1			1		
< = 1.76	33	1.99	1.08–3.67	0.028	1.47	0.73–2.94	0.282
Age at dx (in years)							
> = 2	101	1			1		
0–1	10	0.37	0.13–1.04	0.060	0.46	0.13–1.67	0.239
WBC (× 10^3^/MicroL)							
≤ 100	88	1			1		
> 100	23	2.23	1.10–4.52	0.026	0.76	0.28–2.01	0.576
Cytomolecular risk							
Standard	30	1			1		
Low	63	0.23	0.11–0.45	< 0.001	0.22	0.09–0.49	< 0.001
High	18	0.94	0.42–2.14	0.889	0.80	0.31–2.05	0.646
Multivariable with CM-induced pY-STAT5							
CM-induced pY-STAT5 ∆MFI							
1.18–2.25	71	1			1		
< = 1.18	28	3.10	1.61–5.99	0.001	2.79	1.31–5.95	0.008
> 2.25	12	3.65	1.66–8.02	0.001	2.01	0.72–5.60	0.182
Age at dx (in years)							
> = 2	101	1			1		
0–1	10	0.33	0.12–0.92	0.035	0.38	0.10–1.40	0.145
WBC (× 10^3^/MicroL)							
≤ 100	88	1			1		
> 100	23	2.32	1.15–4.66	0.019	0.96	0.36–2.58	0.938
Cytomolecular risk							
Standard	30	1			1		
Low	63	0.19	0.09–0.39	< 0.001	0.22	0.10–0.51	< 0.001
High	18	0.84	0.38–1.86	0.672	0.81	0.32–2.04	0.652

While the empirically determined cutpoints differed somewhat between signaling parameters, we saw substantial overlap in the lists of patients in the low-response groups. There were 14 patients in both the low G-CSF-induced pY-STAT3 (*n* = 21) and the low CM-induced pY-STAT3 (*n* = 33) response groups and 24 patients in both the low CM-induced pY-STAT3 and the low CM-induced pY-STAT5 response groups (*n* = 30). We were particularly intrigued by the 11 patients (~ 10%) who belonged to all 3 low-response groups. These patients and their clinical characteristics are presented in Table 3. Notably, only 3 (27%) had not experienced an event at last follow-up. Seven of the 8 patients with an event experienced relapse or refractory disease.

**Table 3:** Patients included in all 3 low-response groups (*n* = 11)

USI	Age (years)	Sex	BM blast%	Cytogenetics	FLT3	Mutations	Risk assignment	SCT in CR1?	First event
PANXWX	7.5921	F	81	*t*(9;11) ( +)	WT	None	Standard	Unknown	Induction failure
PAPBEJ	1.0048	F	50	del(7q)( +)	WT	None	Standard	No	Relapse
PARWXU	1.526	M	91% (PB)	del(11p)	WT	None	Standard	No	Relapse
PARXMP	1.463	M	35	Complex; NUP98-KDM5A	WT	None	Standard	No	Censored
PASLTF	16.8569	M	81	NK	WT	CEBPα	Low	No	Censored
PASMYS	2.1027	F	47	+ 8	WT	NPM1, WT1, KIT	Low	No	Relapse
PASXDS	15.6797	M	37	NK	PM	NPM1	Low	No	Censored
PASXVC	20.3751	M	71	NK	ITD 0.37	NPM1	Low	Yes	Relapse
PASYDA	13.9329	M	32	NK	ITD 0.19	NPM1	Low	Yes	Death in CR
PASYWA	14.1848	M	95	NK	ITD 0.93	None	High	Unknown	Induction failure
PATGIY	5.321	M	66	*t*(6;11)	WT	None	Standard	Yes	Relapse

*USI* unique subject identifier, *WT* wild-type, *ITD* internal tandem duplication, *PM* point mutation, *SCT* stem cell transplant, *CR1* first complete remission

### Cases with low pY-STAT3/5 response phenotypes had increased expression of genes encoding hematopoietic factors and respiratory chain proteins

Existing RNA-seq data were available for 68 of the 113 cases (60%) used for the survival analyses. We identified 371 transcripts that were differentially expressed in samples with G-CSF-induced pY-STAT3 ∆MFI ≤ 1.5 compared to samples with ∆MFI > 1.5, at FDR = 0.05 (Online Resource Table S4). All but 39 were upregulated in the low-response group. Similarly, we identified 350 transcripts that were differentially expressed in the low CM-induced pY-STAT3 group, compared to the samples in the higher response group (Online Resource Table S5). All but 11 were higher in the low-response group. We identified 302 transcripts that were differentially expressed between the low and intermediate CM-induced pY-STAT5 groups, with all but 19 higher in the low-response group (Online Resource Table S6). The transcriptomes of the high and intermediate CM-induced pY-STAT5 groups were very similar, with only 2 differentially expressed transcripts identified.

After excluding transcripts that did not map to a known gene, we identified 87 genes that were commonly upregulated in the low G-CSF-induced pY-STAT3 and the low CM-induced pY-STAT3 response groups, compared to their corresponding high-response groups (Online Resource Table S7). We identified 152 genes that were commonly differentially expressed in the CM-induced pY-STAT3 and CM-induced pY-STAT5 response comparisons, of which 151 were higher in the low-response groups (Online Resource Table S8). Finally, 42 genes were commonly upregulated in all 3 low-response groups (Table 4). Interestingly, a number of genes encoding hematopoietic soluble factors were commonly upregulated in all 3 low-response groups, including *CSF2* (GM-CSF), *CSF3* (G-CSF), *CCL20* (MIP-3α) and *CCL22* (MDC). This result is not explained by a difference in the proportion of non-malignant cells in the low-responding samples since the blast percentages were similar between groups (Online Resource Table S2). Notably, 3 components of the mitochondrial electron transport chain (ETC) complex I (*NDUFA3, NDUFA6, NDUFA13*) were increased in all 3 low-response groups. Ingenuity Pathway Analysis of the 42 common genes identified functions related to proliferation, inflammation and transcription (Online Resource Table S9). String, a protein-based algorithm, also identified networks with hematopoietic factors and respiratory chain proteins at the hubs (Online Resource Fig. S4).

**Table 4:** Commonly upregulated genes in all 3 low-response groups

Gene	G-CSF-induced pY-STAT3		CM-induced pY-STAT3		CM-induced pY-STAT5	
	FDR	Log2FC (low/high)	FDR	Log2FC (low/high)	FDR	Log2FC (low/mid)
*ADIRF*	< 1e-4	6.5744	< 1e-4	5.9897	0.001	5.6125
*ANKRD37*	0.015	1.7141	0.0023	1.8159	0.0085	1.6901
*ANOS1*	0.0014	5.2994	0.0043	5.1466	< 1e-4	5.916
*BCYRN1*	< 1e-4	4.3908	< 1e-4	4.3853	0.0023	3.6507
*CAVIN3*	0.0032	4.688	0.027	4.1479	4.00E-04	4.5154
*CCL20*	< 1e-4	6.9573	< 1e-4	6.3836	< 1e-4	6.2951
*CDC42BPA*	0.0055	4.422	6.00E-04	3.7621	0.0085	3.4334
*CDO1*	4.00E-04	3.7092	0.0049	3.2727	0.0195	3.1027
*CENPW*	8.00E-04	1.53	0.0316	1.0592	0.0362	1.1481
*CSF2*	< 1e-4	5.2173	4.00E-04	4.8279	0.0068	3.9251
*CSF3*	< 1e-4	8.3887	< 1e-4	7.8638	3.00E-04	7.3808
*CSKMT*	2.00E-04	2.6544	< 1e-4	2.7212	< 1e-4	2.7129
*DKK1*	0.0222	4.7557	0.0013	4.3437	< 1e-4	5.9058
*DNAAF1*	< 1e-4	7.4279	< 1e-4	6.796	< 1e-4	6.5581
*ELOB*	0.0089	1.3216	0.0109	1.1885	0.0456	1.1028
*ESRRG*	2.00E-04	7.4727	8.00E-04	6.3846	0.0417	6.4541
*HILPDA*	< 1e-4	3.8202	< 1e-4	3.3193	0.0011	3.2191
*HPSE2*	0.0301	7.8146	< 1e-4	8.5754	< 1e-4	8.5329
*HSD11B1-AS1*	< 1e-4	3.9508	0.0083	3.1905	0.0217	3.0611
*IL23A*	< 1e-4	5.2571	< 1e-4	4.8305	1.00E-04	4.5209
*IL4I1*	< 1e-4	3.1947	1.00E-04	2.4834	0.0054	2.219
*IL6*	< 1e-4	4.8538	0.0011	4.1393	0.0109	3.9563
*IL6R-AS1*	< 1e-4	4.2896	0.0011	3.4554	0.0099	3.1758
*LY6K*	0.0397	2.9021	0.0291	2.9798	0.0393	2.8443
*LYPD3*	< 1e-4	3.4425	2.00E-04	2.9239	0.0071	2.5933
*NDUFA13*	0.0184	1.1661	0.0294	1.028	0.0193	1.1314
*NDUFA3*	0.0133	1.5326	0.036	1.3075	0.0098	1.525
*NDUFA6*	0.0154	1.0439	0.0487	0.8535	0.0417	0.9218
*PNMA5*	4.00E-04	4.196	0.0086	3.6158	0.0251	3.513
*PPP1R17*	< 1e-4	6.1809	0.0043	5.0882	0.0484	4.3184
*PPP1R27*	1.00E-04	4.9516	0.0077	4.3288	0.0023	4.3348
*PTGES*	< 1e-4	4.6081	0.0062	3.4467	0.0307	3.1158
*SNHG25*	0.0301	1.5205	0.0322	1.4285	0.0366	1.4536
*ST6GALNAC5*	0.0031	4.9639	< 1e-4	4.651	0.0039	4.1388
*STARD10*	0.0093	1.3999	0.0044	1.3516	0.0328	1.194
*TEX15*	0.0032	6.4781	0.0059	3.7189	0.013	3.7723
*TNFAIP6*	< 1e-4	2.8495	0.0011	2.3017	0.0099	2.1905
*TNFRSF9*	5.00E-04	2.4794	0.0158	1.8904	0.0307	1.8416
*TRPC6*	0.0156	7.4776	< 1e-4	6.0442	< 1e-4	5.7396
*VTRNA1-3*	0.0019	3.4363	0.0011	3.4755	0.0219	2.8717
*YJEFN3*	0.0236	1.0885	0.0316	0.9716	0.0215	1.0585
*ZNF687-AS1*	0.0026	1.8023	0.017	1.5161	0.0364	1.4661
Top upstream regulators						
Upstream regulator		Predicted state		Activation *z*-score		*p* overlap
CD36		Activated		2		1.38E-06
IL1B		Activated		2.104		2.38E-08
IL17A		Activated		2.122		2.54E-07
NFKBIZ		Activated		2.177		4.56E-11
TNF		Activated		2.372		3.13E-04

Using the upstream regulator function of the Ingenuity Pathway Analysis [28], we identified upstream regulatory programs that could account for the gene signature associated with low environment-induced STAT3 and STAT5 activation. Activation of pro-inflammatory cytokines (TNFα, IL-1β and IL-17A) and their downstream mediators (NFKBIZ) are statistically associated with the observed low STAT3/5 response gene expression profile (Table 4). Consistent with the increased expression of ETC complex 1 genes, we further identified CD36 as a likely upstream regulator. CD36 is a scavenger receptor that takes up long-chain fatty acids to fuel mitochondrial fatty acid oxidation in chemoresistant AML cells [29, 30]. Therefore, our findings suggest that pediatric AML cases with low inducible STAT3/5 may derive their chemotherapy resistance from specific inflammatory interactions and energy production processes.

### Genes highly expressed by low STAT3/5 response cases were associated with poor prognosis in an independent pediatric AML dataset

**Fig. 4:**
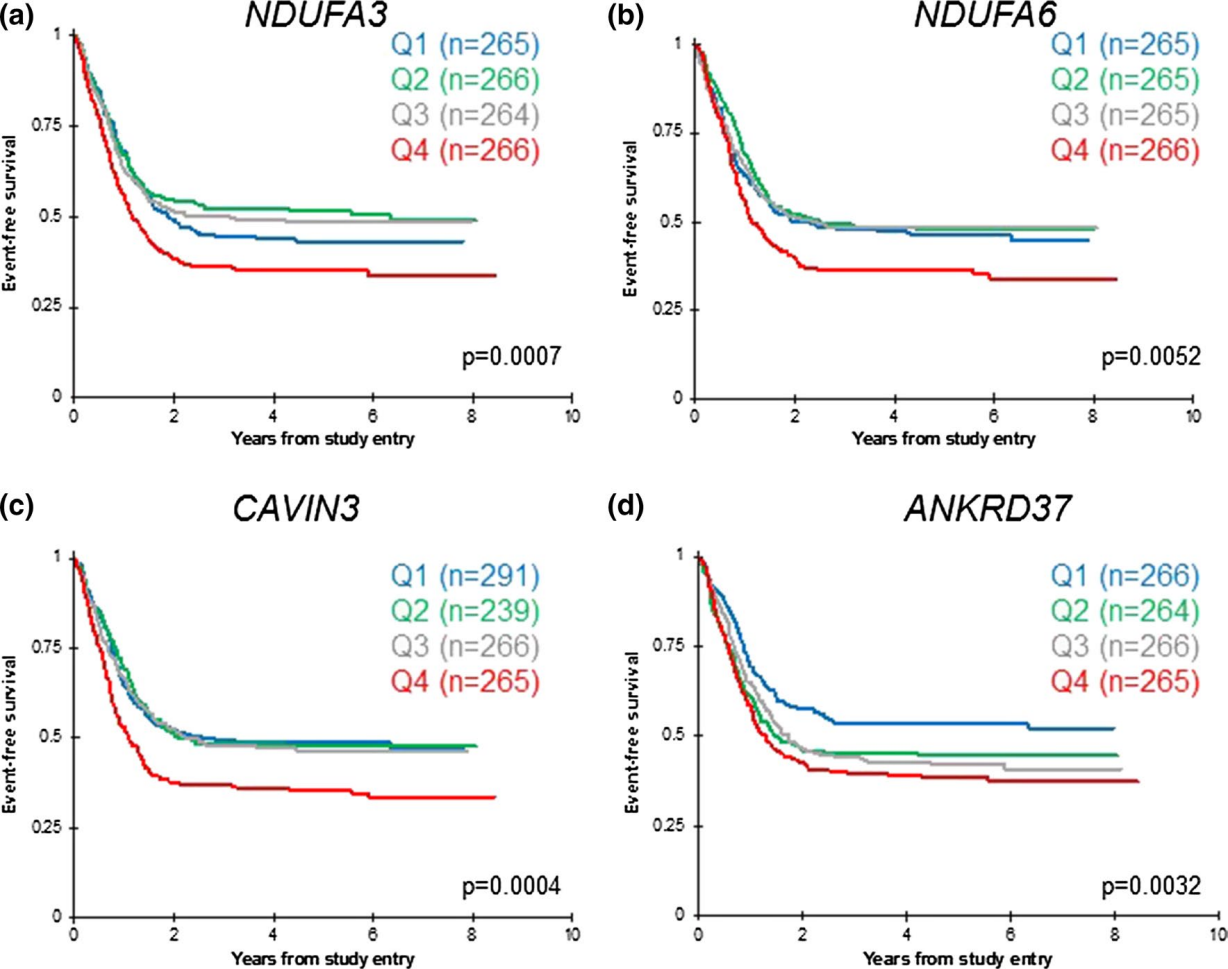
Genes from the 42-gene low-response signature have prognostic value in pediatric AML. **a**, **b** Two genes encoding respiratory chain complex I subunits, *NDUFA3* and *NDUFA6*, demonstrated significantly worse EFS for patients with expression levels in the highest quartile (Q4), compared to those with lower expression. **c** Patients with expression of the signaling regulator *CAVIN3* in the highest quartile (Q4) had significantly lower EFS than those in quartiles 1–3. **d** Patients with expression of the HIF1α target *ANKRD37* in quartiles 2–4 had significantly inferior outcomes compared to patients with expression in the lowest quartile (Q1). Kaplan–Meier estimates are based on clinically annotated RNA-seq data for 1061 patients in the TpAML dataset. Log-rank *p*-values are shown

To evaluate the prognostic relevance of genes that were overexpressed in the low-response groups, we queried the TpAML dataset, which includes RNA-seq data for 1061 pediatric AML patients who enrolled in the COG phase III trial AAML1031 [3]. Two of the three genes encoding respiratory chain complex I subunits, *NDUFA3* and *NDUFA6*, demonstrated significantly worse EFS for patients with higher expression compared to those with lower expression (Fig. 4a, b). Patients with higher expression of *CAVIN3*, which encodes a caveolae-associated protein that regulates endocytosis, had significantly inferior EFS (Fig. 4c). Patients with higher expression of *ANKRD37*, a target gene of the hypoxia sensor HIF1α, had significantly inferior outcomes compared to patients with lower expression (Fig. 4d). These transcriptome comparisons reveal candidate genes for further studies that will elucidate mechanisms of aberrant environment-mediated signaling and of treatment failure.

## Discussion

In this study of pediatric AML patients enrolled on contemporary COG trials, we demonstrated that aberrantly low STAT3 and STAT5 responses to multiple environment-derived factors are associated with inferior EFS. Patients whose AML cells demonstrated aberrantly low STAT3 activation upon stimulation with stromal cell CM had inferior outcomes compared to patients with a stronger inducible STAT3 response. Likewise, patients with aberrantly low CM-induced pY-STAT5 had significantly inferior EFS. The prognostic relevance of CM-induced STAT3/5 activity retained significance in multivariate modeling taking into account cytomolecular risk, age, and presenting WBC. Therefore, the phenotype of low environment-induced STAT-pathway signaling is a novel and independent functional prognostic factor in pediatric AML.

A recent study of inducible STAT3/5 signaling in pediatric AML patients by Schumich, et al., did not find a relationship between G-CSF-induced pY-STAT3 and outcome [23]. Importantly, their survival analysis did not include patients classified as low risk, whereas in our study, it was those patients with low-risk cytogenetics who tended to have high G-CSF-induced pY-STAT3 (∆MFI > 1.5; Online Resource Table S2). The same study reported that higher GM-CSF-induced pY-STAT5 was associated with higher relapse risk. We found that very high and very low CM-induced pY-STAT5 responses were associated with poor EFS. Schumich, et al., also measured pY-STAT5 induced by a cytokine cocktail, but they did not report on any relationships between those responses and outcome.

The association between STAT3/5 activation and EFS also has been studied in adult AML patients. Notably, the results for pediatric patients are the opposite of those reported for adult AML patients [10, 11]. The difference in signaling pathway biology between adults and children is yet another example of how pediatric AML is different from adult AML. Thousands of adult and pediatric AML cases have been characterized at the molecular level, revealing numerous differences between age groups [14, 31–34]. Our results show that adult and pediatric AML cells also differ functionally.

Further, our results suggest that mechanisms that blunt STAT3 signaling also blunt STAT5, and these mechanisms are independent of the ligand-receptor interaction itself. The IL-6, IL-3 and GM-CSF receptors are comprised of alpha and beta subunits that colocalize with downstream mediators in specialized membrane domains containing clathrin, caveolin, or specific lipids (e.g. lipid rafts). Internalization of the ligand-receptor complex is an important step in signaling and also regulates receptor surface expression (as recently reviewed [35, 36]). Defective membrane domain organization or endocytosis could explain intact receptor surface expression but decreased signaling activity downstream of multiple ligands, as we saw in our samples.

The dysfunction that accounts for low inducible STAT3/5 signaling could also contribute to poor outcomes. For instance, extrinsic apoptosis signaling also requires organization of signaling components in lipid rafts, so a generalized lipid raft dysfunction could explain aberrantly low signaling and aberrantly low apoptosis. Another explanation could be that AML cells that activate STAT3/5 are more proliferative, whereas AML cells that do not activate STAT3/5 are more quiescent and thereby resistant to drugs that kill dividing cells. Further studies are required to determine the mechanisms explaining the relationship between signaling responses and patient outcomes.

We recognize that CM-induced signaling responses are not likely to be developed for clinical testing, but a corresponding gene signature could more readily be clinically validated. We identified a number of genes that were differentially expressed between patients in the low-response groups compared to those in the higher response groups. Interestingly, several genes encoding hematopoietic factors were paradoxically upregulated in the low-response groups. It is possible that the feedback pathways that normally limit factor expression are not engaged when ligand-induced signaling is impaired. Several genes encoding adhesion signaling components also were upregulated in the low-response groups. Adhesion to the microenvironment is a well-known mechanism by which malignant cells derive protection from cytotoxic chemotherapy [37].

To unify the differentially expressed genes into physiological processes, we identified their common upstream regulators. Here, multiple pro-inflammatory factors—TNFα, IL-1β, IL-17A, and NFKBIZ—were revealed to be potential drivers of the gene expression profile associated with low inducible STAT3/5. This finding is important in several ways. First, expression of these inflammatory mediators by AML cells is driven by their adhesion to stromal cells [38, 39], reinforcing the concept that cases with low inducible STAT3/5 signaling are likely to derive their chemoresistance from alternative, contact-dependent survival pathways. Second, IL-17A not only drives inflammation but also recruits myeloid-derived suppressor cells [40], and thus may contribute to the poor outcomes of these patients by promoting immune evasion. Third, these inflammatory pathways are targetable. Secukinumab is a monoclonal antibody against IL-17A that is FDA-approved for the treatment of psoriasis. TNF inhibitors, including etanercept, infliximab and adalimumab, are FDA-approved for the treatment of multiple inflammatory conditions like rheumatoid arthritis.

We also identified metabolism genes among those upregulated in cases with low STAT3/5 activation. Specifically, genes encoding ETC complex 1 proteins were expressed more highly in those cases. Additionally, CD36 was identified as a likely upstream regulator. CD36 is a fatty acid translocase that takes up substrates for mitochondrial fatty acid oxidation and is expressed on leukemia stem cells [29]. Several recent studies have demonstrated that AML cells that survive chemotherapy depend on oxidative phosphorylation [30, 41]. Indeed, AML cells that survived cytarabine had increased expression of CD36, and blocking fatty acid oxidation with etomoxir sensitized AML cells to cytarabine-induced apoptosis in vitro [30]. Other drugs that interfere with oxidative phosphorylation have shown promise in preclinical [42] and clinical studies [43].

## Conclusion

Our data demonstrate that aberrant inducible STAT3/5 signaling identifies a subset of pediatric AML patients with inferior EFS, independent of cytomolecular features. AML cells from these patients are further characterized by a specific gene expression profile that suggests an inflammatory bone marrow microenvironment and dependence on oxidative metabolism. Taken together, our results reveal potential mechanisms of chemotherapy resistance that can be targeted to improve outcomes.

## Supplementary Information

Below is the link to the electronic supplementary material.

Supplementary file1 (PDF 544 KB)

Supplementary file2 (XLS 256 KB)

## Data Availability

Existing RNA-seq data were obtained from the NCI Therapeutically Applicable Research to Generate Effective Treatments (TARGET) database, available at https://portal.gdc.cancer.gov/projects. Additional RNA-seq data were obtained from the Target Pediatric AML (TpAML) project (targetpediatricaml.org). These data will be publicly available in dbGap by spring 2021. Other data from the current study are available from the corresponding author on reasonable request.
