# Supplementary material for: Aberrantly low STAT3 and STAT5 responses are associated with poor outcome and an inflammatory gene expression signature in pediatric acute myeloid leukemia
Source: Clin Transl Oncol. 2021 May 4;23(10):2141–54. doi: 10.1007/s12094-021-02621-w (PMC8390401; doi:10.1007/s12094-021-02621-w)
Supplement: Supplementary file 1 — Supplementary file1 (PDF 544 KB) [file 12094_2021_2621_MOESM1_ESM.pdf]

**Supplemental Table S1. Soluble factor levels in AML bone marrow and HS5 CM**			
Soluble Factor	AML BM plasma – Minimum (pg/ml)	AML BM plasma – Maximum (pg/ml)	50% HS5 CM ± SD (pg/ml)
EGF	4.6	343.4	5.7 ± 1.2
Eotaxin	21.9	327.2	22.1 ± 14.4
FGF-2	16.5	681.4	41.6 ± 13.8
Flt3L	2.09	119.2	20.3 ± 11.1
Fracktalkine	7.1	1072	173.1 ± 49.5
G-CSF	3.4	9386	7741.4 ± 4981.8
GM-CSF	2.8	2425	207.1 ± 121.0
GRO	16.4	13741	1303.9 ± 841.4
IFNα	2.8	475.6	25.4 ± 14.2
IFNγ	3.8	1033	9.2 ± 5.5
IL-1α	3.7	1325	12.0 ± 6.9
IL-1β	1.4	10709	2.2 ± 1.6
IL-2	1.1	159.1	5.4 ± 5.4
IL-3	0.6	208.0	0.7 ± 0.2
IL-4	3.2	140.5	20.6 ± 12.5
IL-5	1.2	241.9	1.7 ± 0.5
IL-6	1.6	12059	2890.4 ± 2340.5
IL-7	1.8	26.0	24.6 ± 10.5
IL-8	*12.0*	*4202*	*9820 ± 3217.8*
IL-9	1.0	55.9	3.9 ± 2.3
IL-10	1.7	267.3	6.3 ± 5.7
IL-12p40	1.8	534.7	25.0 ± 24.3
IL-12p70	2.6	389.4	4.3 ± 3.5
IL-13	0.5	618.2	10.4 ± 8.0
IL-15	0.6	59.0	5.5 ± 3.2
IL-17α	1.2	346.1	2.3 ± 1.4
IL-1RA	11.9	5785	32.1 ± 25.0
IP-10	127.3	12324	690.3 ± 356.0
MCP-1	*22.9*	*5078*	*8134 ± 4734.2*
MCP-3	*1.6*	*2057*	*5660.1 ± 4748.8*
MDC	*67.1*	*2411*	*18.3 ± 11.7*
MIP-1α	2.9	7758	11.7
MIP-1β	2.3	1335	10.3 ± 7.0
PDGF-AA	103.1	4126	137.8 ± 9.2
PDGF-BB	*350.2*	*9170*	*74.2 ± 82.1*
RANTES	*999.8*	*13229*	*163.2 ± 66.5*
sCD40L	*70.5*	*16415*	*51.8 ± 35.7*
TGFα	*2.4*	*204.6*	*1.7*
TNFα	*4.8*	*1376*	*2.2 ± 0.8*
TNFβ	1.7	737.5	8.0 ± 8.9
VEGF	34.3	2834	513.7 ± 57.8

Factor levels were measured with the Milliplex Human Cytokine Magnetic Bead 41-plex kit. (EMD Millipore) for diagnostic bone marrow plasma from pediatric AML patients (n=31) and for HS5 CM (n=3 replicates). These results have been reported in part previously, and detailed methods are found within those publications (see references 19 and 20). Factors for which the average 50% CM level is outside the range for AML bone marrow plasma are indicated with italics.

Supplemental Table S2. Patient Characteristics for pY-STAT3 Response Groups

	G-CSF-induced pY-STAT3 ∆MFI ≤1.5		G-CSF-induced pY-STAT3 ∆MFI >1.5				CM-induced pY-STAT3 ∆MFI ≤1.76		CM-induced pY-STAT3 ∆MFI >1.76		
Characteristic	N	%	N	%	p		N	%	N	%	p
Total	24	21.6%	87	78.4%			33	29.2%	80	70.8%	
**Study**											
AAML03P1	3	12.5%	12	13.8%	1.000		5	15.2%	10	12.5%	0.763
AAML0531	21	87.5%	75	86.2%			28	84.8%	70	87.5%	
Arm A	3	14.3%	42	56.0%	**<0.001**		11	39.3%	34	48.6%	0.405
Arm B	18	85.7%	33	44.0%			17	60.7%	36	51.4%	
**Gender**											
Male	14	58.3%	43	49.4%	0.440		23	69.7%	35	43.8%	**0.012**
Female	10	41.7%	44	50.6%			10	30.3%	45	56.3%	
**Age (years) at diagnosis**											
0-1 yo	5	20.8%	5	5.7%	**0.037**		6	18.2%	4	5.0%	0.061
2-10 yo	7	29.2%	34	39.1%	0.373		12	36.4%	29	36.3%	0.991
≥ 11 yo	12	50.0%	48	55.2%	0.653		15	45.5%	47	58.8%	0.197
Median (range)	10.8	(1.0 - 20.7)	11.8	(0.6 - 28.7)	0.328		8.4	(0.6 - 20.4)	12.1	(1.2 - 28.7)	**0.047**
**Race**											
American/Alaskan Native	0	0.0%	1	1.3%	1.000		0	0.0%	1	1.4%	1.000
Asian	0	0.0%	2	2.5%	1.000		1	3.1%	1	1.4%	0.527
Black or African American	4	18.2%	6	7.5%	0.217		5	15.6%	5	7.0%	0.278
White	18	81.8%	71	88.8%	0.470		26	81.3%	64	90.1%	0.217
Unknown	2		7				1		9		
**Ethnicity**											
Hispanic	7	30.4%	10	12.0%	0.051		7	21.2%	10	13.3%	0.390
Not Hispanic	16	69.6%	73	88.0%			26	78.8%	65	86.7%	
Unknown	1		4				0		5		
**Cytogenetics**											
Normal	7	29.2%	25	29.4%	0.981		14	42.4%	19	24.4%	0.057
t(8;21)	1	4.2%	13	15.3%	0.297		1	3.0%	14	17.9%	**0.036**
inv(16)	1	4.2%	23	27.1%	**0.017**		4	12.1%	20	25.6%	0.114
KMT2A-rearranged	5	20.8%	7	8.2%	0.132		5	15.2%	7	9.0%	0.336
t(6;9)	0	0.0%	3	3.5%	1.000		0	0.0%	3	3.8%	0.553
monosomy 7	0	0.0%	1	1.2%	1.000		0	0.0%	1	1.3%	1.000
del(7q)	1	4.2%	1	1.2%	0.394		1	3.0%	1	1.3%	0.508
monosomy 5/del(5q)	1	4.2%	0	0.0%	0.220		0	0.0%	1	1.3%	1.000

+8	2	8.3%	3	3.5%	0.303		2	6.1%	3	3.8%	0.633
Other	6	25.0%	9	10.6%	0.091		6	18.2%	9	11.5%	0.356
Unknown	0		2				0		2		
**French-American-British morphology**											
M0	2	11.8%	0	0.0%	**0.029**		1	3.7%	1	1.4%	0.473
M1	0	0.0%	13	16.3%	0.116		5	18.5%	8	11.1%	0.334
M2	1	5.9%	25	31.3%	**0.036**		3	11.1%	25	34.7%	**0.020**
M4	6	35.3%	30	37.5%	0.864		11	40.7%	25	34.7%	0.579
M5	4	23.5%	8	10.0%	0.215		4	14.8%	8	11.1%	0.731
M6	1	5.9%	0	0.0%	0.175		1	3.7%	0	0.0%	0.273
M7	1	5.9%	0	0.0%	0.175		0	0.0%	1	1.4%	1.000
Other	2	11.8%	4	5.0%	0.282		2	7.4%	4	5.6%	0.663
Unknown	7		7				6		8		
**FLT3/ITD status**											
Positive	5	20.8%	19	22.1%	0.895		7	21.2%	17	21.5%	0.971
Negative (WT)	19	79.2%	67	77.9%			26	78.8%	62	78.5%	
Unknown	0		1				0		1		
**CEBPα status**											
Mutated	1	4.2%	10	11.8%	0.450		3	9.1%	8	10.3%	0.851
Wild-type	23	95.8%	75	87.2%			30	90.9%	70	88.6%	
Unknown	0		2				0		2		
**NPM status**											
Mutated	5	20.8%	11	12.9%	0.339		8	24.2%	8	10.3%	0.076
Wild-type	19	79.2%	74	87.1%			25	75.8%	70	89.7%	
Unknown	0		2				0		2		
**WT1 status**											
Mutated	1	4.2%	8	9.4%	0.681		2	6.1%	7	9.0%	0.723
Wild-type	23	95.8%	77	90.6%			31	93.9%	71	91.0%	
Unknown	0		2				0		2		
**Cytomolecular Risk[1]**											
Standard	12	50.0%	17	20.0%	**0.003**		14	42.4%	16	20.5%	**0.018**
Low	8	33.3%	54	63.5%	**0.008**		16	48.5%	47	60.3%	0.253
High	4	16.7%	14	16.5%	1.000		3	9.1%	15	19.2%	0.185
Unknown	0		2				0		2		
**MRD at End Induction 1**											

Positive[2]	5	26.3%	23	31.9%	0.636		5	19.2%	24	36.4%	0.111
Negative	14	73.7%	49	68.1%			21	80.8%	42	63.6%	
Unknown	5		15				7		14		
**Morphology at End Induction 1**											
CR[3]	15	62.5%	69	81.2%	0.055		28	84.8%	58	74.4%	0.227
Not CR	9	37.5%	16	18.8%			5	15.2%	20	25.6%	
Unevaluable	0		2				0		2		
**WBC at study entry ($x10^3$/MicroL)**											
<100	20	83.3%	67	77.0%	0.505		28	84.8%	61	76.3%	0.310
≥100	4	16.7%	20	23.0%			5	15.2%	19	23.8%	
BM Blast% at study entry Median (range)	71	(32 - 100)	70	(3 - 100)	0.736		66.2	(23 - 100)	71	(3 - 100)	0.668
Blood Blast% at study entry Median (range)	45	(0 - 93)	51	(0 - 97)	0.273		40	(0 - 95)	56	(0 - 97)	0.054
**Outcomes**											
5 year EFS from study entry[4]	29.2 ± 18.6		56.4 ± 10.8		**0.044**		36.4 ± 16.7		57.2 ± 11.4		0.051
5 year OS from study entry[4]	54.2 ± 20.3		68.4 ± 10.3		0.244		57.1 ± 17.4		69.2 ± 10.8		0.172

1. Cytomolecular risk groups: Low: t(8;21), inv(16), $NPM1^{mut}$, $CEBP\alpha^{mut}$; High: monosomy 5/7, FLT3/ITD with allelic ratio > 0.4; Standard: no low or high risk criteria with known cytogenetics
2. Minimal residual disease by flow cytometry, positive: ≥0.02%
3. CR: complete remission with <5% blasts by morphology
4. Percent ± 2SE% with log-rank p-values

Supplemental Table S3. Patient Characteristics for pY-STAT5 Response Groups							
	CM-induced pY-STAT5 ∆MFI ≤1.18		CM-induced pY-STAT5 ∆MFI 1.19-2.25		CM-induced pY-STAT5 ∆MFI >2.25		
Characteristic	N	%	N	%	N	%	p
Total	28	24.8%	73	64.6%	12	10.6%	
**Study**							
AAML03P1	3	10.7%	10	13.7%	2	16.7%	0.865
AAML0531	25	89.3%	63	86.3%	10	83.3%	
Arm A	10	40.0%	30	47.6%	5	50.0%	0.782
Arm B	15	60.0%	33	52.4%	5	50.0%	
**Gender**							
Male	16	57.1%	37	50.7%	5	41.7%	0.657
Female	12	42.9%	36	49.3%	7	58.3%	
**Age (years) at diagnosis**							
0-1 yo	6	21.4%	4	5.5%	0	0.0%	**0.024**
2-10 yo	9	32.1%	28	38.4%	4	33.3%	0.824
≥ 11 yo	13	46.4%	41	56.2%	8	66.7%	0.466
Median (range)	8.3	(0.6 - 20.4)	11.9	(1.2 - 28.7)	13.7	(5.9 - 22.5)	0.079
**Race**							
American/Alaskan Native	0	0.0%	1	1.6%	0	0.0%	0.735
Asian	1	3.7%	1	1.6%	0	0.0%	0.696
Black or African American	3	11.1%	7	10.9%	0	0.0%	0.482
White	23	85.2%	55	85.9%	12	100.0%	0.373
Unknown	1		9		0		
**Ethnicity**							
Hispanic	7	25.0%	7	10.3%	3	25.0%	0.128
Not Hispanic	21	75.0%	61	89.7%	9	75.0%	
Unknown	0		5		0		
**Cytogenetics**							
Normal	12	42.9%	19	26.8%	2	16.7%	0.166
t(8;21)	1	3.6%	12	16.9%	2	16.7%	0.205
inv(16)	3	10.7%	17	23.9%	4	33.3%	0.206
KMT2A-rearranged	3	10.7%	8	11.3%	1	8.3%	0.955
t(6;9)	0	0.0%	2	2.8%	1	8.3%	0.328
monosomy 7	0	0.0%	0	0.0%	1	8.3%	**0.016**
del(7q)	1	3.6%	1	1.4%	0	0.0%	0.678
monosomy 5/del(5q)	0	0.0%	1	1.4%	0	0.0%	0.753

+8	2	7.1%	2	2.8%	1	8.3%	0.514
Other	6	21.4%	9	12.7%	0	0.0%	0.195
Unknown	0		2		0		
**French-American-British morphology**							
M0	1	4.3%	1	1.5%	0	0.0%	0.627
M1	5	21.7%	7	10.8%	1	9.1%	0.374
M2	4	17.4%	21	32.3%	3	27.3%	0.393
M4	7	30.4%	24	36.9%	5	45.5%	0.687
M5	3	13.0%	8	12.3%	1	9.1%	0.944
M6	1	4.3%	0	0.0%	0	0.0%	0.188
M7	0	0.0%	1	1.5%	0	0.0%	0.768
Other	2	8.7%	3	4.6%	1	9.1%	0.706
Unknown	5		8		1		
**FLT3/ITD status**							
Positive	6	21.4%	13	17.8%	5	45.5%	0.114
Negative (WT)	22	78.6%	60	82.2%	6	54.5%	
Unknown	0		0		1		
**CEBPα status**							
Mutated	3	10.7%	8	11.1%	0	0.0%	0.510
Wild-type	25	89.3%	64	87.7%	11	100.0%	
Unknown	0		1		1		
**NPM status**							
Mutated	6	21.4%	10	13.9%	0	0.0%	0.225
Wild-type	22	78.6%	62	86.1%	11	100.0%	
Unknown	0		1		1		
**WT1 status**							
Mutated	2	7.1%	6	8.3%	1	9.1%	0.973
Wild-type	26	92.9%	66	91.7%	10	90.9%	
Unknown	0		1		1		
**Cytomolecular Risk[1]**							
Standard	13	46.4%	15	21.1%	2	16.7%	**0.027**
Low	13	46.4%	44	62.0%	6	50.0%	0.328
High	2	7.1%	12	16.9%	4	33.3%	0.116
Unknown	0		2		0		
**MRD at End Induction 1**							
Positive[2]	5	21.7%	18	31.6%	6	50.0%	0.233

Negative	18	78.3%	39	68.4%	6	50.0%	
Unknown	5		16		0		
**Morphology at End Induction 1**							
CR[3]	25	89.3%	54	76.1%	7	58.3%	0.089
Not CR	3	10.7%	17	23.9%	5	41.7%	
Unevaluable	0		2		0		
**WBC at study entry (x10^3/MicroL)**							
<100	25	89.3%	55	75.3%	9	75.0%	0.292
≥100	3	10.7%	18	24.7%	3	25.0%	
BM Blast% at study entry Median (range)	66	(31 - 96)	72	(3 - 100)	70	(36 - 100)	0.623
Blood Blast% at study entry Median (range)	53	(0 - 95)	48.7	(0 - 97)	59	(28 - 88.5)	0.646
**Outcomes**							
5 year EFS from study entry[4]	28.6 ± 17.1		62.8 ± 11.6		33.3 ± 27.2		**<0.001**
5 year OS from study entry[4]	45.5 ± 19.1		75.0 ± 10.6		58.3 ± 28.5		**0.009**

1. Cytomolecular risk groups: Low: t(8;21), inv(16), NPM1^{mut}, CEBPα^{mut} ; High: monosomy 5/7, FLT3/ITD with allelic ratio > 0.4; Standard: no low or high risk criteria with known cytogenetics
2. Minimal residual disease by flow cytometry, positive: ≥0.02%
3. CR: complete remission with <5% blasts by morphology
4. Percent ± 2SE% with log-rank p-values

Supplemental Table S9. Top Enriched Functions*		
**Categories**	**Diseases or Functions Annotation**	**p-value**
Cellular Growth and Proliferation,Lymphoid Tissue Structure and Development	Proliferation of lymphatic system cells	3.67E-06
Cellular Development,Cellular Growth and Proliferation	Proliferation of blood cells	8.98E-06
Cellular Development,Cellular Growth and Proliferation,Hematological System Development and Function,Lymphoid Tissue Structure and Development	Proliferation of mononuclear leukocytes	3.26E-05
Cellular Movement	Invasion of tumor cell lines	2.32E-04
Cancer,Organismal Injury and Abnormalities,Reproductive System Disease	Breast or ovarian cancer	2.07E-03
Cancer,Organismal Injury and Abnormalities,Reproductive System Disease	Breast cancer	5.56E-03
Cellular Development,Cellular Growth and Proliferation	Cell proliferation of tumor cell lines	6.66E-03
Cell Death and Survival	Cell viability	8.81E-03
Cancer,Neurological Disease,Organismal Injury and Abnormalities	High grade astrocytoma	8.96E-03
Cancer,Neurological Disease,Organismal Injury and Abnormalities	Glioma	8.97E-03
Inflammatory Disease	Chronic inflammatory disorder	9.50E-03
Cancer,Organismal Injury and Abnormalities	Breast or pancreatic cancer	1.60E-02
Cancer,Endocrine System Disorders,Organismal Injury and Abnormalities,Reproductive System Disease	Ovarian cancer	1.62E-02
Gene Expression	Transcription	1.65E-02
Cancer,Hematological Disease,Organismal Injury and Abnormalities	Mature lymphocytic neoplasm	1.87E-02
Gene Expression	Transcription of DNA	2.16E-02

*Functions with >5 genes in the category

Supplemental Figure S1

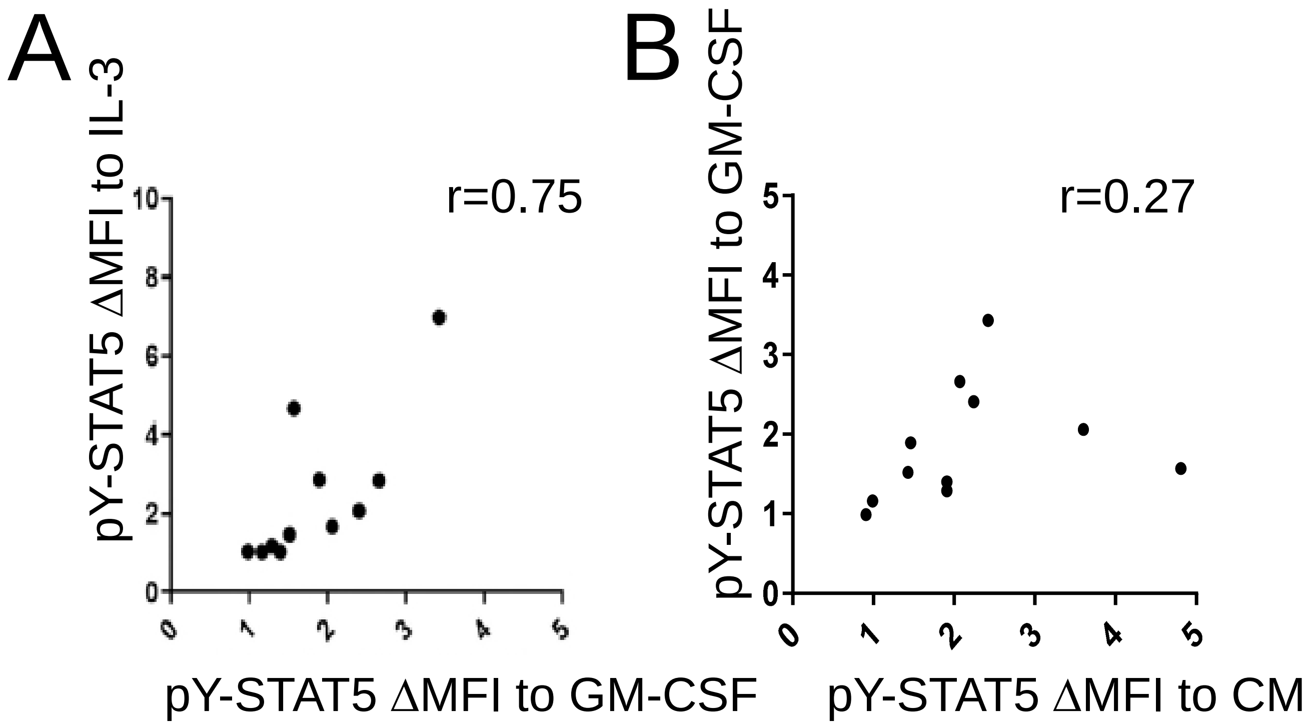

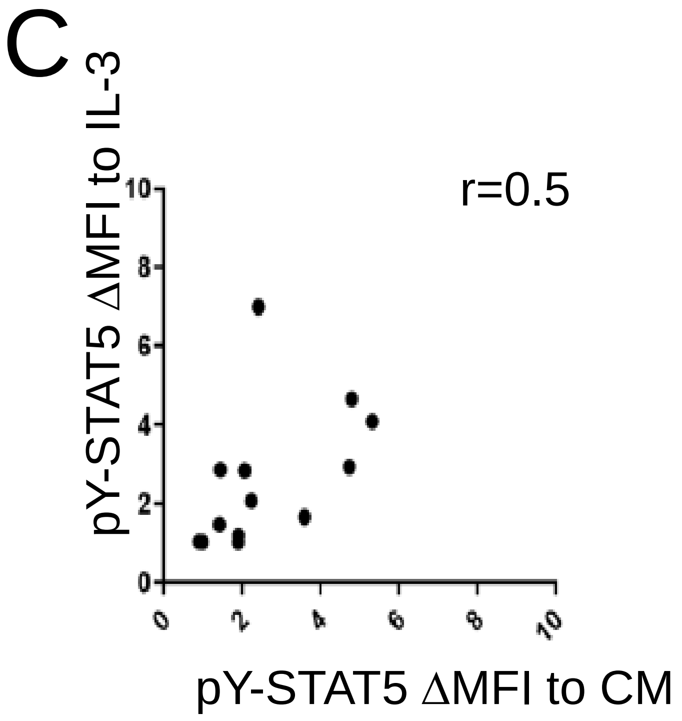

Figure S1. STAT5 responses to GM-CSF and IL-3 were strongly correlated with each other but not with STAT5 responses to CM. (A) The pY-STAT5 ΔMFIs in response to GM-CSF and IL-3, which use the same CD131 signal transducing subunit, were strongly correlated (n=11). The pY-STAT5 ΔMFIs in response to **(B)** GM-CSF and **(C)** IL-3 were not related to the responses to the cocktail of factors present in CM (n=11-13). r = Pearson correlation coefficient.

Supplemental Figure S2

A

	G-CSF ΔMFI	IL-6 ΔMFI	CM ΔMFI
Basal pY-STAT3 MFI	-0.18	-0.22	-0.20
Basal pY-STAT5 MFI	-0.33	-0.48	-0.10
Basal pERK1/2 MFI	-0.25	-0.09	-0.24

B

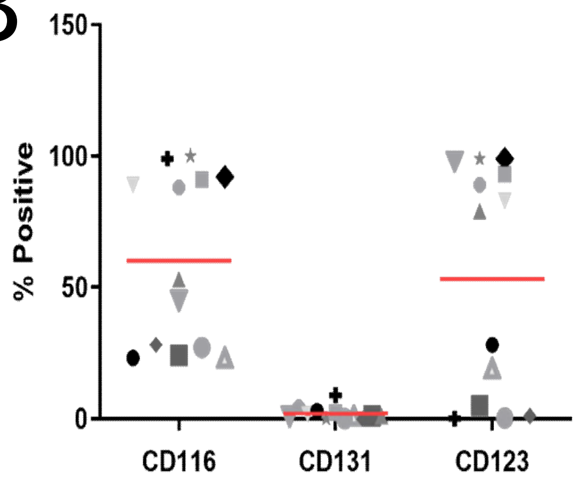

C

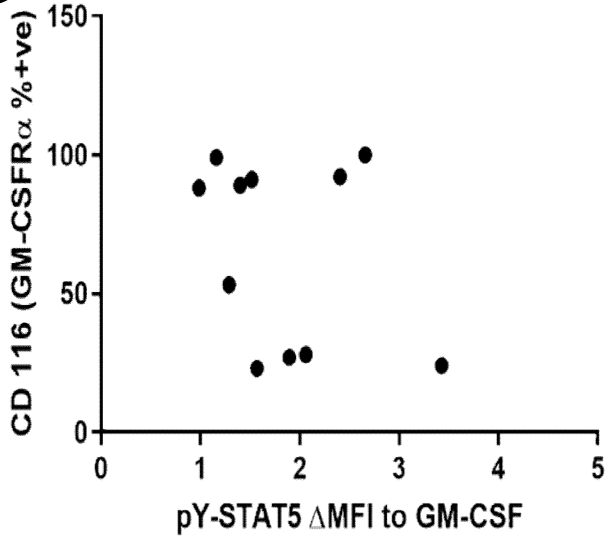

D

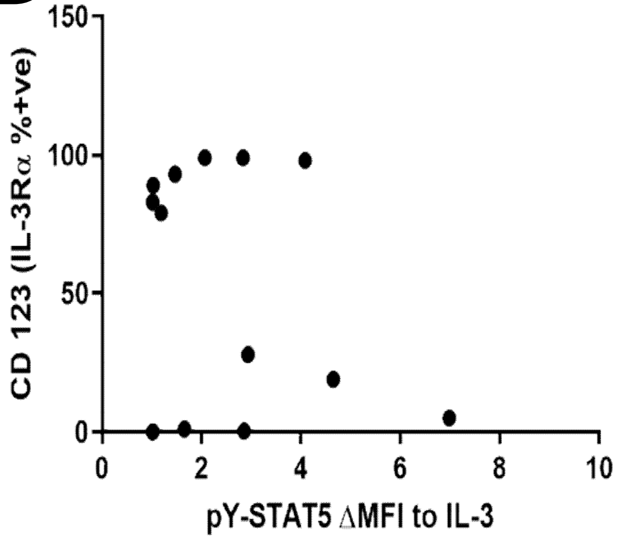

Figure S2. Dysfunctional STAT3/5 activation is not due to elevated basal activation or to absence of surface receptors. (A) The heatmap illustrates the degree of correlation for each ligand-induced ΔMFI and its corresponding basal MFI. Numbers represent the Pearson correlation coefficient (r). (**B)** Primary AML samples were stained for the CD116 (GM-CSF receptor alpha), CD123 (IL-3 receptor alpha), and CD131 (common beta receptor). The percent of events in the positive region are shown (n=13). **(C, D)** Ligand-induced pY-STAT5 ΔMFIs were not related to the expression of the ligand's receptor (n=11-13).

Supplemental Figure S3

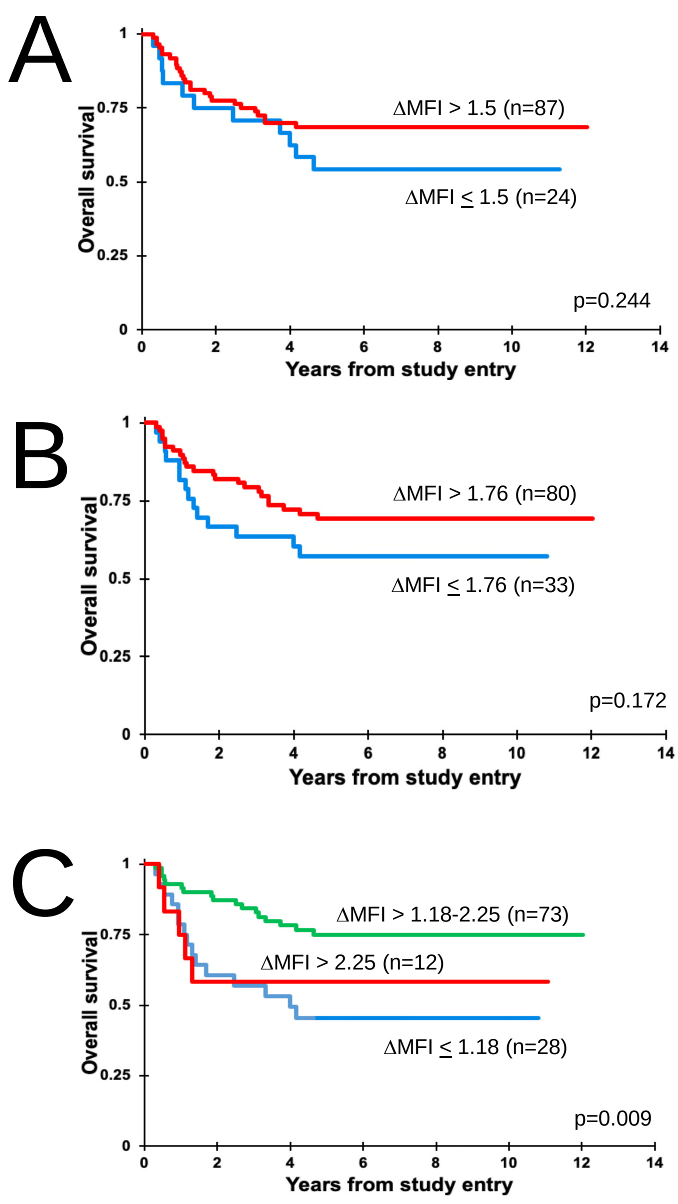

Figure S3. Inducible pY-STAT5, but not pY-STAT3, was associated with overall survival. (A) Patients whose AML cells had a G-CSF-induced pY-STAT3 DMFI ≤ 1.5 had a 5-year OS of 54.2 ± 20.3% compared to 68.4 ± 10.3% for patients with a DMFI > 1.5 (p=0.244). **(B)** Patients whose AML cells had a CM-induced pY-STAT3 DMFI ≤ 1.76 had a 5-year OS of 57.1 ± 17.4% v. 69.2 ± 10.8% for those with DMFI > 1.76 (p=0.172). **(C)** Patients with CM-induced pY-STAT5 DMFI ≤ 1.18 and patients with CM-induced pY-STAT5 DMFI > 2.25 had 5-year OS of 45.5 ± 19.1% and 58.3 ± 28.5% respectively, compared to the patients in the intermediate response group (75.0 ± 10.6%; (p=0.009).

Supplemental Figure S4

A

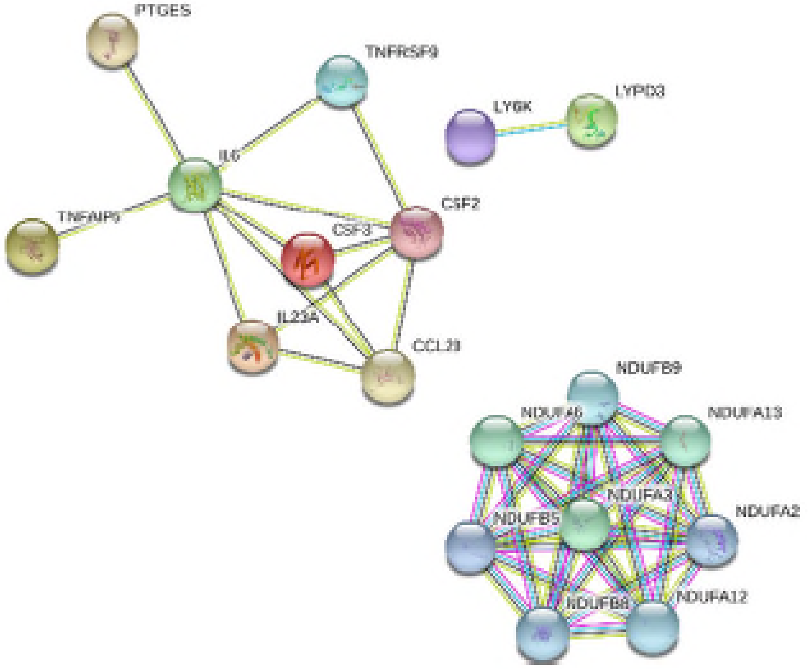

B

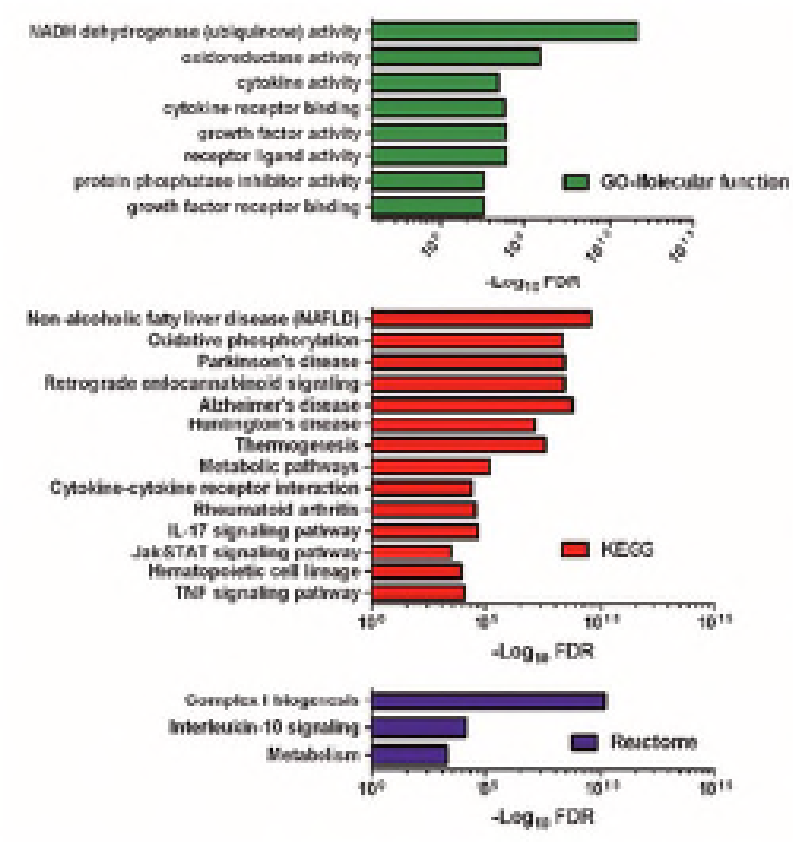

Figure S4. STRING analysis of enriched functions in the low pY-STAT3/5 response groups. (A) The genes that were commonly upregulated in all 3 low response groups (Table 3) were used as query in STRING 11.0 to visualize the predicted protein-protein interactions (PPI). The network interaction has a PPI enrichment p-value of 2.77 x 10^{-11}. **(B)** Functional pathway enrichment analysis was performed using the STRING 11.0 protein-based algorithm. The pathway enrichment data from KEGG, Reactome pathways, and GO annotation for molecular functions are presented with $-\text{Log}_{10}$ FDR.
